# Pro-Angiogenic Response to OsteoBiol^®^ GTO^®^ in an In Vitro Endothelial Cell Model Is Associated with Modulation of the COX-2/PGE2/VEGF Axis

**DOI:** 10.3390/bioengineering13080907

**Published:** 2026-08-11

**Authors:** Alessia Ricci, Tea Romasco, Marwa Balaha, Adriano Piattelli, Amelia Cataldi, Natalia Di Pietro, Susi Zara

**Affiliations:** 1 Department of Pharmacy, “G. d’Annunzio” University of Chieti-Pescara, 66100 Chieti, Italy; alessia.ricci@unich.it (A.R.); marwa.balaha@pharm.tanta.edu.eg (M.B.); cataldi@unich.it (A.C.); s.zara@unich.it (S.Z.); 2 Department of Medical, Oral and Biotechnological Sciences, “G. d’Annunzio” University of Chieti-Pescara, 66100 Chieti, Italy; tea.romasco@unich.it; 3 Department of Pharmaceutical Chemistry, Faculty of Pharmacy, Kafrelsheikh University, Kafrelsheikh 33516, Egypt; 4 School of Dentistry, UniCamillus-Saint Camillus International University of Health and Medical Sciences, 00131 Rome, Italy; apiattelli51@gmail.com; 5 Facultad de Medicina, UCAM Universidad Católica San Antonio de Murcia, 30107 Murcia, Spain; 6 Department of Neurosciences, Imaging and Clinical Sciences, “G. d’Annunzio” University of Chieti-Pescara, 66100 Chieti, Italy

**Keywords:** Osteobiol^®^ GTO^®^, endothelial cells, angiogenesis, COX-2, PGE2, VEGF, bone regeneration, dental biomaterials, collagen

## Abstract

Alveolar bone resorption after tooth extraction complicates subsequent dental implant placement. OsteoBiol^®^ GTO^®^ (Tecnoss^®^, Giaveno, Italy) is an innovative pre-hydrated heterologous collagenated bone mix blended with a thermosensitive copolymer (OsteoBiol^®^ TSV Gel) that has demonstrated osteoconductive properties. Despite direct contact with blood vessels upon socket filling, its pro-angiogenic potential has not been directly investigated on endothelial cells. Thus, in this study, an in vitro model, consisting of the EA.hy926 endothelial cell line exposed to different OsteoBiol^®^ GTO^®^ soaking preparations [original soaking (OS), centrifuged soaking (CS), and diluted soaking (DS)] at multiple concentrations (1, 5, 10, and 20 mg/mL) was established to identify optimal experimental conditions and characterize the underlying molecular mechanisms. Cell viability and collagen release quantification led to the selection of 10 mg/mL OS as the most suitable condition. Under this condition, OsteoBiol^®^ GTO^®^ induces an early increase in Cyclooxygenase-2 (COX-2) protein expression and Prostaglandin E-2 (PGE2) secretion, followed by upregulation of Vascular Endothelial Growth Factor (VEGF) protein expression and phosphorylation of Endothelial Nitric Oxide Synthase (eNOS) at serine 1177. The tube formation assay confirmed the pro-angiogenic functional outcome. These results suggest an association between the pro-angiogenic response of endothelial cells to OsteoBiol^®^ GTO^®^ and modulation of the COX-2/PGE2/VEGF axis. This effect could be attributed to collagen accumulation in the OS, thereby representing a novel and promising pro-angiogenic mechanism with potential implications for wound healing and guided bone regeneration following tooth extraction.

## 1. Introduction

Tooth extraction is among the most common dental procedures, and it inevitably triggers a cascade of biological events culminating in remodeling and partial resorption of the alveolar socket. After extraction, the loss of mechanical stimulation from the periodontal ligament initiates osteoclast-mediated bone resorption, which, if left unmanaged, results in significant horizontal and vertical bone loss in the first months after surgery [1]. This collapse of both hard and soft tissues compromises the anatomical prerequisites for subsequent osseointegrated dental implant placement, often necessitating complex and costly bone augmentation procedures [2,3,4].

To preserve alveolar ridge dimensions and maintain an environment suitable for implantation, socket preservation techniques using bone substitutes and barrier membranes have been extensively investigated over the past two decades [5,6,7]. Among the biomaterials developed for this purpose, xenogeneic and alloplastic substitutes have gained widespread clinical acceptance for their osteoconductive scaffolding properties, safety profile, and availability [8]. Nevertheless, an ideal socket filler should not only support osteogenesis but also promote vascularization, as angiogenesis is a prerequisite for bone formation and remodeling [9,10,11].

OsteoBiol^®^ GTO^®^ (Tecnoss^®^, Giaveno, Italy) is among the most innovative xenograft biomaterials available. It is a pre-hydrated heterologous collagenated bone mix blended with the thermosensitive copolymer OsteoBiol^®^ TSV Gel (Tecnoss^®^, Giaveno, Italy). OsteoBiol^®^ TSV Gel is a heterologous gel diluted in an aqueous solution containing a biocompatible synthetic copolymer that imparts thermo-reversible and thermo-gelling properties. The granular architecture of OsteoBiol^®^ GTO^®^ closely mimics the structural organization of native bone, rendering it moldable, easily resorbable, and osteoconductive. Additionally, the collagen component promotes stabilization of the blood clot following extraction, thereby creating a favorable early-healing environment [12]. These characteristics have made OsteoBiol^®^ GTO^®^ an attractive candidate for guided bone regeneration (GBR) protocols [13]. Previous in vitro studies have characterized the effects of OsteoBiol^®^ GTO^®^ on cells of the human dental compartment, including osteoblasts [14], periodontal ligament (PDL) cells [15], gingival cells [16], and dental pulp stem cells (hDPSCs) [17,18], consistently demonstrating its ability to support osteogenic differentiation and proliferation. However, no study has yet investigated the direct effect of OsteoBiol^®^ GTO^®^ on endothelial cells. This represents a significant knowledge gap, since, upon socket filling, OsteoBiol^®^ GTO^®^ is in direct contact with remnant blood vessels. The modulation of endothelial behavior by the biomaterial—and, in particular, the stimulation of a pro-angiogenic response—could profoundly influence the quality of bone regeneration, as vascularization is required to supply oxygen, nutrients, and osteogenic progenitors to the regenerating tissue [19,20,21]. The only existing studies examining an OsteoBiol^®^ GTO^®^–endothelial interaction employed an indirect model in which conditioned medium from injured PDL or human gingival cells was first obtained in the presence of OsteoBiol^®^ GTO^®^ and then applied to human umbilical vein endothelial cells (HUVECs), precluding conclusions about the direct action of the biomaterial on the vascular compartment [15,16].

In this context, the present study was designed to: (i) establish and optimize an in vitro model to test OsteoBiol^®^ GTO^®^ directly on the EA.hy926 endothelial cell line, systematically comparing different soaking preparation methods and concentrations; (ii) quantify collagen released from OsteoBiol^®^ GTO^®^ soaking preparations; and (iii) investigate the molecular mechanisms underlying the pro-angiogenic response elicited by OsteoBiol^®^ GTO^®^ in endothelial cells, with a particular focus on the Cyclooxygenase-2/Prostaglandin E-2/Vascular Endothelial Growth Factor (COX-2/PGE2/VEGF) signaling pathway.

## 2. Materials and Methods

### 2.1. Cell Culture

The human umbilical vein cell line EA.hy926 was purchased from ATCC (LGC Standards S.r.l., Milan, Italy) and cultured in Dulbecco’s Modified Eagle Medium (DMEM) with 4.5 g/L glucose, supplemented with 10% Fetal Bovine Serum (FBS), 1% penicillin/streptomycin, and 1% L-glutamine, all purchased from EuroClone S.p.A (Milan, Italy). Cells were subcultured and maintained at 37 °C in a humidified 5% CO_2_ atmosphere.

### 2.2. Soaking Preparation and Cell Treatments

**Figure 1:**
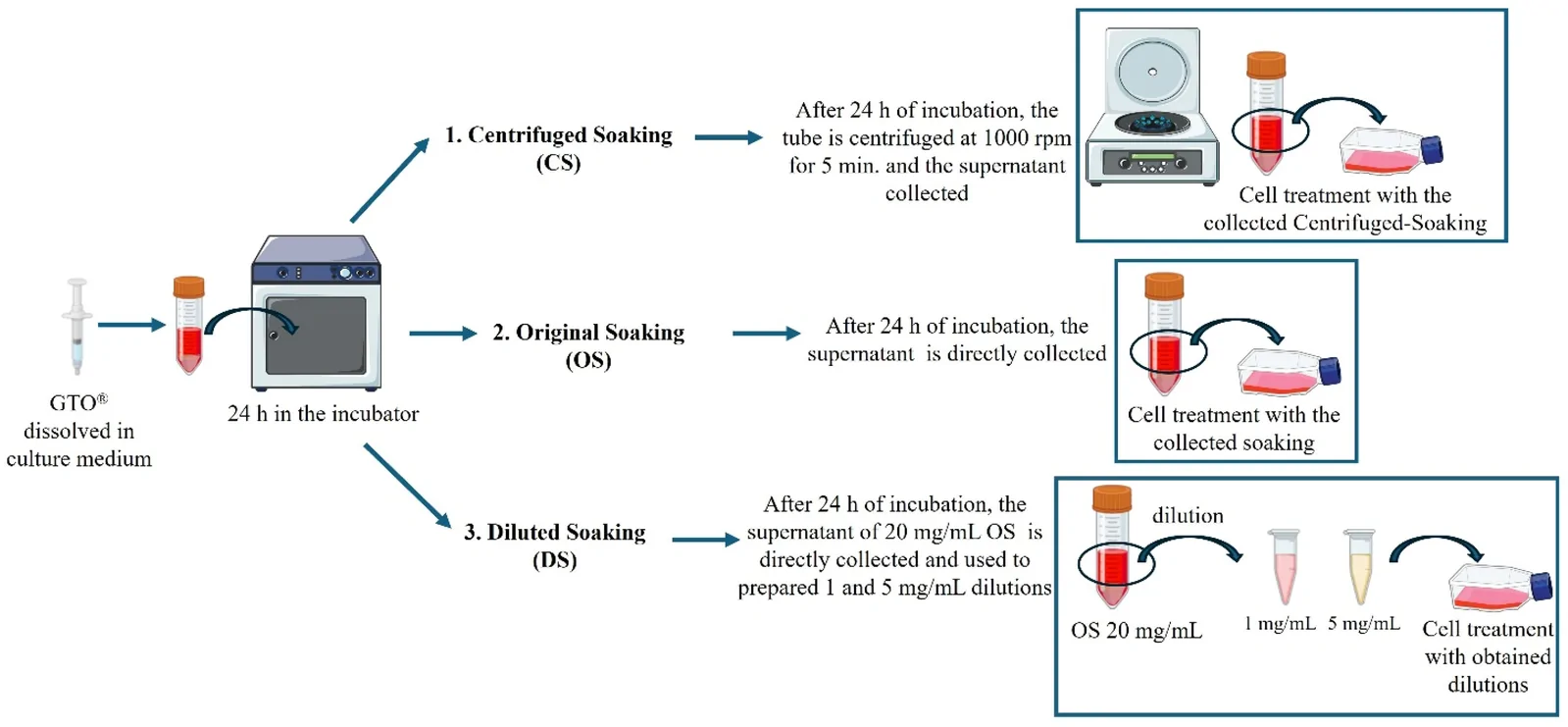
Procedural explanation of three soaking preparation methods: original soaking (OS), centrifuged soaking (CS), and diluted soaking (DS).

OsteoBiol^®^ GTO^®^ (Tecnoss^®^, Giaveno, Italy) is an innovative, pre-hydrated, heterologous, collagenated bone mix (with particles ranging from 600 to 1000 µm) blended with a thermosensitive copolymer (OsteoBiol^®^ TSV Gel). It is commercially available in pre-filled syringes. OsteoBiol^®^ GTO^®^ was prepared at concentrations of 1, 5, 10, and 20 mg/mL for soaking. Specifically, OsteoBiol^®^ GTO^®^ was weighed on a precision balance (previously sterilized under UV light) within a laminar flow hood and incubated in complete medium at 37 °C for 24 h. Three soaking preparation methods were tested on EA.hy926, adapted from protocols described in the literature for analogous biomaterials [14,15,17,18]:

1. Original Soaking (OS): After 24 h of incubation, the supernatant above the OsteoBiol^®^ GTO^®^ granules was collected and directly applied to the cells.
2. Centrifuged Soaking (CS): After 24 h of incubation, the conditioned medium was centrifuged at 1000 rpm for 5 min to compact OsteoBiol^®^ GTO^®^ granules and disperse collagen throughout the medium. The resulting supernatant was collected and applied to the cells.
3. Diluted Soaking (DS): From the 20 mg/mL OS, two dilutions were prepared and used for treatment: 1:20 and 1:4 to obtain 1 and 5 mg/mL concentrations, respectively (Figure 1).

At the predetermined experimental times, samples were collected for further analysis.

### 2.3. Cell Metabolic Activity Assay (MTT)

EA.hy926 cells were seeded in 96-well tissue-treated plates at 8000 cells/well. The following day, cells were treated with the obtained OsteoBiol^®^ GTO^®^ OS, CS (at 1, 5, 10, and 20 mg/mL), and DS (at 1 and 5 mg/mL) to determine the optimal working dose and settings. After 24 h and 48 h of OsteoBiol^®^ GTO^®^ treatment, cells were subjected to a metabolic activity test: the culture medium was removed from each well and replaced with a solution containing 0.5 mg/mL 3-(4,5-dimethylthiazol-2-yl)-2,5-diphenyltetrazolium bromide (MTT). During the subsequent 4 h of incubation, metabolically active cells converted MTT into violet formazan salts. The formazan crystals were then dissolved in dimethyl sulfoxide (DMSO). The absorbance of the final solution was measured spectrophotometrically at 570 nm using a microplate reader (Varioscan Lux, Thermo Scientific, Waltham, MA, USA). Results from three biological replicates were normalized to untreated cell (CTRL) values and expressed as percentages of cell viability.

### 2.4. Quantification of Collagen Released

Collagen release from the various OsteoBiol^®^ GTO^®^ soaking preparations was evaluated using a modified Sirius Red-based colorimetric assay [22]. Briefly, 1 mL of each soaking preparation (OS, CS, and DS) was transferred to sterile 2 mL Eppendorf tubes. Then, 0.5 mL of 0.1% Direct Red 80 in 3% acetic acid was added to each sample and mixed thoroughly. Samples were incubated at room temperature for 30 min to form collagen–dye complexes. After incubation, samples were centrifuged at 8000× *g* for 6 min to precipitate the collagen-bound dye complex. The supernatant was carefully removed without disturbing the pellet, and the precipitates were washed with 1.5 mL of 0.1 M HCl (Merck, Darmstadt, Germany), followed by another centrifugation under the same conditions. After removing the wash, the collagen-bound dye was eluted with 1 mL of 0.1 M NaOH (Merck, Darmstadt, Germany). The resulting eluates were transferred to a transparent 96-well plate, and absorbance was measured at 540 nm using a microplate reader (Varioscan Lux, Thermo Fisher Scientific, Waltham, MA, USA). NaOH solution served as the blank control. For collagen quantification, a standard calibration curve was generated from serial dilutions of collagen prepared in DMEM. In addition, the collagen–dye precipitates were examined microscopically using phase-contrast light microscopy.

### 2.5. PGE2 Secretion (ELISA)

After 6 and 24 h of OsteoBiol^®^ GTO^®^ treatment, PGE2 released into the culture medium was quantified using a PGE2 enzyme immunoassay kit (PGE-2 Enzyme Immunoassay Kit ADI-900-001) from Enzo Life Sciences (Farmingdale, NY, USA), as previously described [23]. Absorbance was measured at 405 nm using a Varioscan Lux spectrophotometer (Thermo Fisher Scientific, Waltham, MA, USA). PGE2 secretion levels from three independent experiments were normalized to the optical density (OD) values obtained from the MTT assay.

### 2.6. Protein Extraction and Western Blot Analysis

EA.hy926 cells were seeded in T25 flasks at 500,000 cells/flask and treated with 10 mg/mL OS for 24 and 48 h. After treatment, cells were collected to obtain a protein pool, as described elsewhere [24], and quantified using the bicinchoninic acid assay (QuantiPro™ BCA Assay kit, Merck Life Science, Milan, Italy). Next, 30 µg of protein was loaded onto an SDS-polyacrylamide gel, electrophoresed, and then transferred to nitrocellulose membranes. The membranes were incubated overnight at 4 °C with the primary antibodies listed in Table 1.

The day after, the membranes were incubated with specific IgG horseradish peroxidase (HRP)-conjugated secondary antibodies (Calbiochem, Darmstadt, Germany). Immunoreactive bands were visualized using the ECL detection system and highlighted with the iBright Imaging System 1500 (Thermo Fisher Scientific, Waltham, MA, USA). Densitometric analysis was performed using the iBright Analysis Software, version 5.5.0 (Thermo Fisher Scientific, Waltham, MA, USA), and values were expressed as fold increase relative to CTRL.

### 2.7. RNA Extraction, Reverse Transcription (RT), and Real-Time RT-Polymerase Chain Reaction (RT-PCR)

EA.hy926 cells were seeded in T25 flasks at 500,000 cells/flask and treated with 10 mg/mL OS. After 6 and 14 h of treatment, cell pellets were collected, and total RNA was isolated using the PureLink^®^ RNA Mini Kit (Life Technologies, Carlsbad, CA, USA) according to the manufacturer’s instructions. To remove residual DNA, samples were incubated with 80 µL of DNase solution (PureLink^®^ DNase Treatment, Life Technologies, Carlsbad, CA, USA) for 15 min. The extracted RNA was eluted in 30 µL of nuclease-free water, and its concentration was quantified using the Qubit^®^ RNA BR Assay Kit (Life Technologies, Carlsbad, CA, USA). cDNA synthesis was performed using the High-Capacity cDNA Reverse Transcription Kit (Life Technologies, Carlsbad, CA, USA). mRNA expression levels were measured by real-time PCR using PowerUpTM SYBRTM Green Master Mix (2×) (Thermo Fisher Scientific, Waltham, MA, USA). Each amplification reaction was prepared in a MicroAmp^®^ Optical 96-well Reaction Plate (Life Technologies, Carlsbad, CA, USA) by combining 10 µL of SYBR Green Master Mix, 1 µM each of the forward and reverse primers, 10 ng of cDNA, and nuclease-free water to a final volume of 20 µL. Primer sequences are provided in Table 2.

Quantitative Polymerase Chain Reaction (qPCR) was performed according to the manufacturer’s recommended run protocol on a QuantStudio 3 system (Thermo Fisher Scientific, Waltham, MA, USA). Gene expression data were analyzed using QuantStudio™ Design & Analysis Software v 1.5.1 (Thermo Fisher Scientific, Waltham, MA, USA), and results were evaluated using the ΔΔCt method.

### 2.8. Tube Formation Assay

The tube formation assay was performed by adding 50 µL of ECM Gel (8–12 mg/mL, Merck Life Science, Milan, Italy) to each well of a 96-well plate and allowing it to solidify at 37 °C for 60 min. After incubation, EA.hy926 cells (4 × 10^4^) were resuspended in serum-free medium containing 10 mg/mL OS and seeded into Matrigel-coated wells. After 20 h of OsteoBiol^®^ GTO^®^ treatment, images were acquired using an inverted light microscope (Leica DMI1; Leica Cambridge Ltd., Cambridge, UK) equipped with a Leica MC120 HD camera (Leica Cambridge Ltd., Cambridge, UK). Capillary-like closed structures were observed, and quantification was performed using ImageJ software (version 1.54k) with the Angiogenesis Analyzer plugin, measuring the number of segments, nodes, junctions, and total length in three independent experiments.

### 2.9. Statistical Analysis

Data are presented as mean ± standard deviation (SD) from at least three independent experiments. Statistical comparisons were performed using two-way analysis of variance (ANOVA), followed by Tukey’s post hoc test for multiple comparisons or Student’s *t*-test for pairwise comparisons. Statistical analyses were conducted using GraphPad Prism software (version 9.0; GraphPad Software, San Diego, CA, USA). *p*-values < 0.05 were considered statistically significant.

## 3. Results

### 3.1. Optimization of OsteoBiol^®^ GTO^®^ Soaking Preparation: Cell Viability Assessment

**Figure 2:**
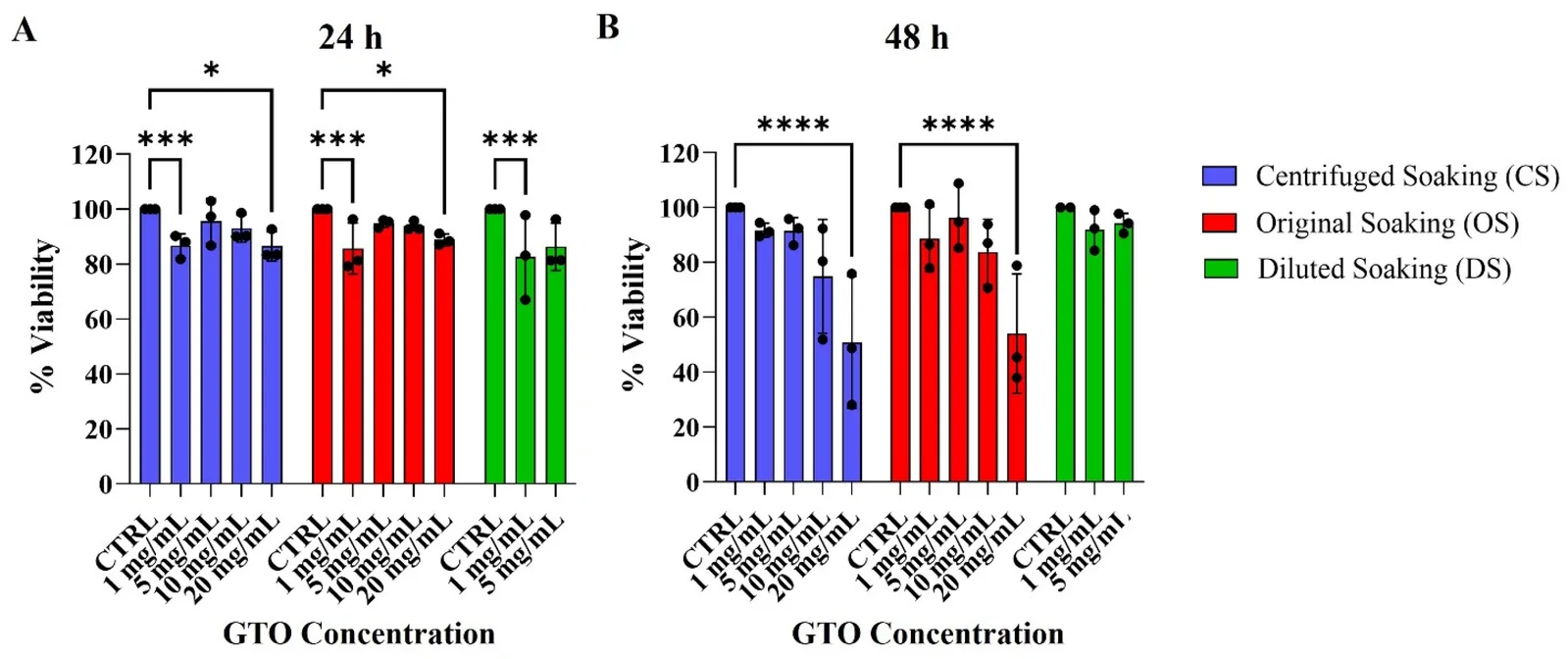
Metabolic activity assay (MTT) performed on EA.hy926 cells after (**A**) 24 h and (**B**) 48 h of treatment with OsteoBiol^®^ GTO^®^ at 1, 5, 10, and 20 mg/mL. CS is shown in blue, OS in red, and DS in green. Metabolic activity of treated samples was normalized to untreated cells (CTRL). The data shown represent the mean ± standard deviation (SD) of three independent experiments. Two-way ANOVA: **** *p* < 0.0001; *** *p* < 0.001; * *p* < 0.05.

The MTT assay was performed to evaluate, after 24 and 48 h of treatment, the effect of OsteoBiol^®^ GTO^®^ at different concentrations (1, 5, 10, 20 mg/mL) prepared by different methods (OS, CS, DS), with the aim of selecting the method that showed the best performance in terms of endothelial cell viability. After 24 h, cells treated with OsteoBiol^®^ GTO^®^ at concentrations of 20 mg/mL and 1 mg/mL across all soaking procedures showed a statistically significant decrease in metabolic activity compared to CTRL cells (Figure 2A); in fact, no statistically significant differences were observed among the soaking types.

Moreover, after 48 h of treatment, cells treated with OsteoBiol^®^ GTO^®^ OS and CS at 20 mg/mL show a statistically significant decrease in viability compared with the CTRL, whereas no statistically significant changes are observed in the presence of OsteoBiol^®^ GTO^®^ OS, CS, and DS at 1 mg/mL and in the CTRL group (Figure 2B). At 5 and 10 mg/mL, cell viability was comparable to that of the CTRL at both experimental time points and across all tested soaking conditions. The MTT assay excluded the 1 and 20 mg/mL concentrations because they were least favorable for cell viability.

### 3.2. Evaluation of Collagen Release from OsteoBiol^®^ GTO^®^ Soaking Preparations

**Figure 3:**
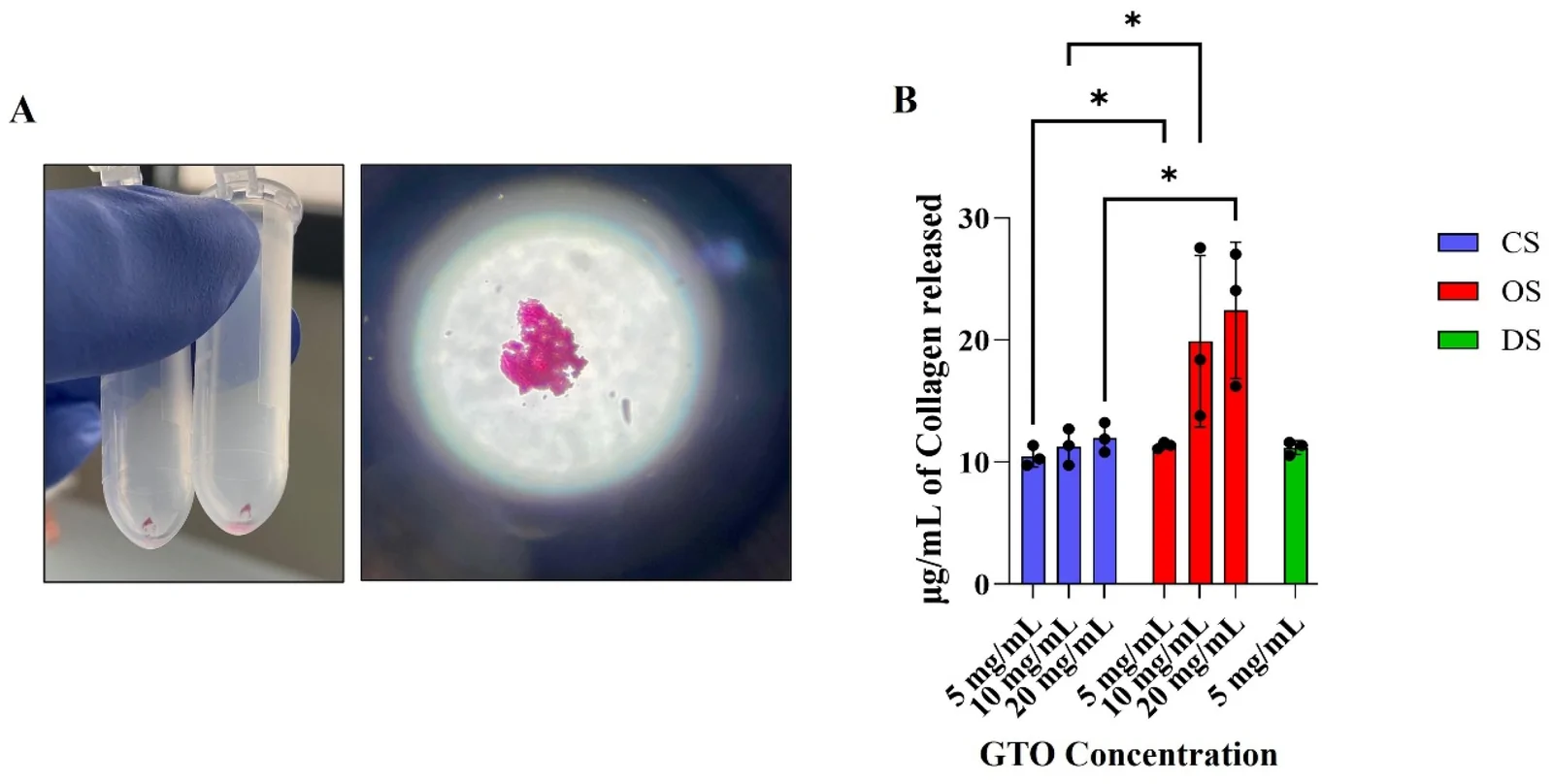
Colorimetric Sirius Red-based assay to determine collagen release in the supernatants after soaking preparations. (**A**) Red pellet (**left** figure) visualized by phase-contrast microscopy (**right**) represents Sirius Red-stained collagen fibers. Magnification: 20×; (**B**) Histogram represents collagen quantification expressed as µg/mL. The data shown represent the mean ± SD from three independent experiments. Two-way ANOVA: * *p* < 0.05.

To evaluate the soaking preparation method in terms of collagen release, the collagen released into the medium after OS, CS, and DS preparation was measured using a colorimetric Sirius Red-based assay (Figure 3A).

Within each group, the amount of collagen detected is dose-dependent (Figure 3B): from 5 mg/mL to 20 mg/mL, collagen concentration increased for both OS and CS. OS at 5, 10, and 20 mg/mL releases a statistically significantly greater amount of collagen than CS at the same concentrations. No statistically significant differences in collagen detection are observed with 5 mg/mL DS compared with either 5 mg/mL OS or CS.

Based on MTT and collagen release results, the OS condition, which yielded the highest collagen release compared with the other OsteoBiol^®^ GTO^®^ preparation methods, and the 10 mg/mL concentration, which proved to be the most suitable for cell viability, were selected as the optimal working conditions for subsequent experiments.

### 3.3. OsteoBiol^®^ GTO^®^ Induces an Early Upregulation of COX-2 Protein Expression and PGE2 Secretion

**Figure 4:**
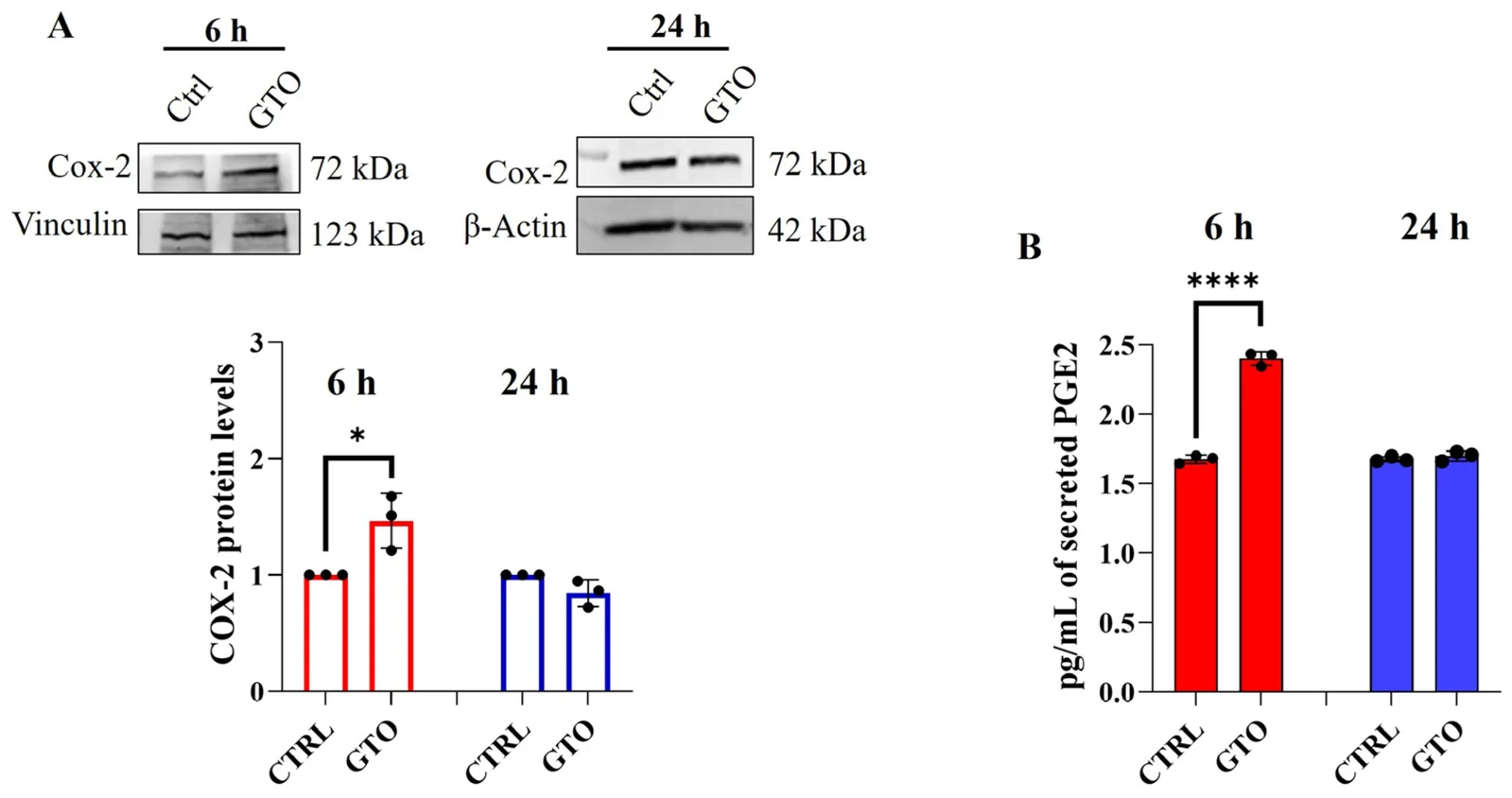
(**A**) Western blot analysis of COX-2 expression after 6 and 24 h of treatment in the presence of OsteoBiol^®^ GTO^®^ OS 10 mg/mL. The histogram displays densitometric values for COX-2 obtained from immunoreactive bands and is expressed as mean ± SD from three independent experiments. Densitometric values were normalized to the loading control (Vinculin) and expressed as fold increase relative to CTRL. Unpaired *t*-test, CTRL vs. OsteoBiol^®^ GTO^®^: * *p* < 0.05. (**B**) ELISA of PGE2 secretion at 6 and 24 h after treatment with OsteoBiol^®^ GTO^®^ OS 10 mg/mL. Secretion levels are reported as pg/mL and normalized to MTT OD viable cells. The data shown represent the mean ± SD from three independent experiments. Unpaired *t*-test CTRL vs. OsteoBiol^®^ GTO^®^: **** *p* < 0.0001.

COX-2 protein expression and PGE2 secretion in the culture medium were evaluated after 6 and 24 h of 10 mg/mL OS treatment to assess their potential role in the pro-angiogenic effect of OsteoBiol^®^ GTO^®^. The results showed a statistically significant increase in COX-2 protein expression after 6 h of treatment (Figure 4A), along with a similarly significant increase in PGE2 secretion, both compared to CTRL (Figure 4B).

COX-2 protein levels return to the basal level of CTRL after a 24 h OsteoBiol^®^ GTO^®^ treatment, with no statistically significant differences between the samples (Figure 4A). Similarly, PGE2 secretion decreases after 24 h of treatment, with no significant differences compared to CTRL (Figure 4B).

### 3.4. OsteoBiol^®^ GTO^®^ Promotes VEGF Expression and Endothelial Nitric Oxide Synthase (eNOS) Phosphorylation on Ser1177

**Figure 5:**
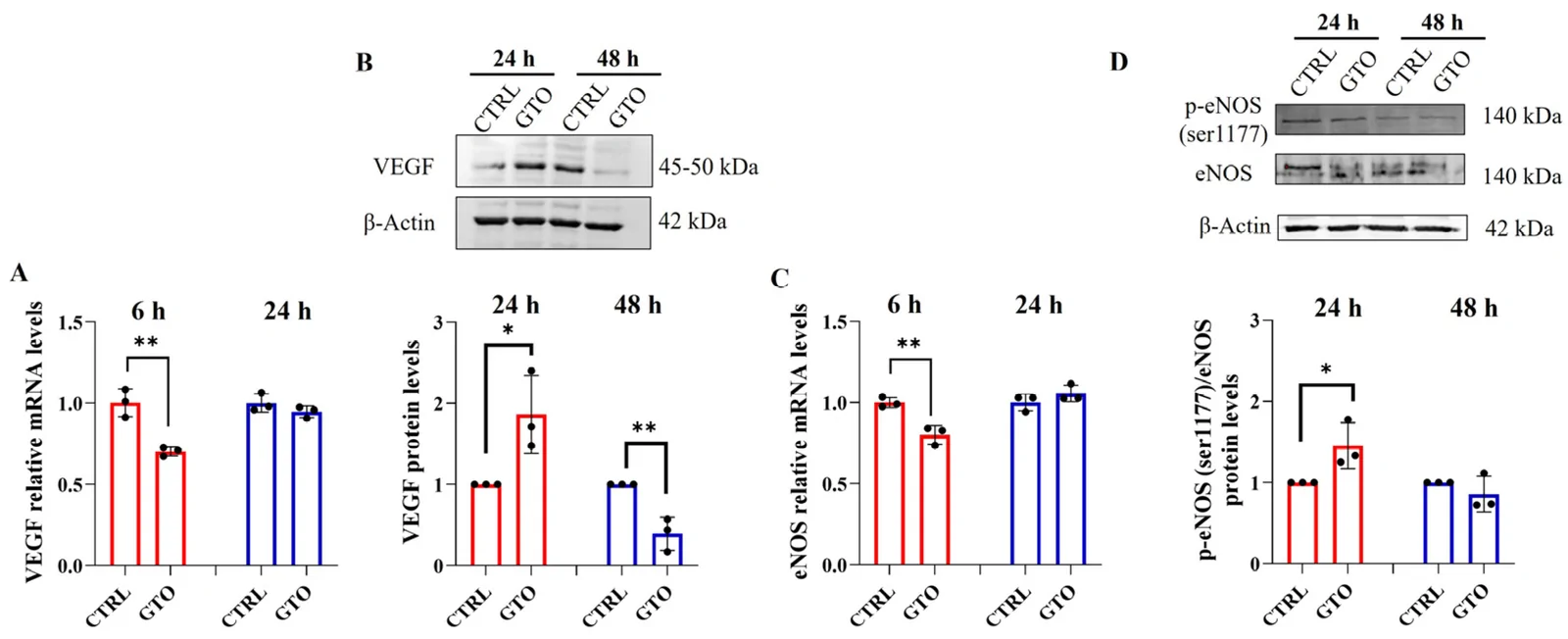
Relative gene expression of (**A**) VEGF and (**C**) eNOS after 6 and 24 h of treatment with OsteoBiol^®^ GTO^®^ OS 10 mg/mL. Data are expressed relative to CTRL (calibrator sample, defined as 1). The data shown represent the mean ± SD from three independent experiments. Unpaired *t*-test CTRL vs. OsteoBiol^®^ GTO^®^: ** *p* < 0.01. Western blot analysis of (**B**) VEGF and (**D**) p-eNOS (Ser1177)/eNOS ratio after 24 and 48 h of OsteoBiol^®^ GTO^®^ OS 10 mg/mL treatment. Histograms display densitometric values obtained from immunoreactive bands and are expressed as the mean ± SD of three independent experiments. Densitometric values were normalized to the loading control (Actin) and expressed as fold increase relative to CTRL. Unpaired *t*-test, CTRL vs. OsteoBiol^®^ GTO^®^: * *p* < 0.05.

VEGF and Endothelial Nitric Oxide Synthase (eNOS) activation through Ser1177 phosphorylation are two downstream targets of the COX-2/PGE2 pathway. Real-time PCR analysis of VEGF and eNOS gene expression shows that, after 6 h in the presence of OsteoBiol^®^ GTO^®^, both VEGF (Figure 5A) and eNOS (Figure 5C) mRNA levels are consistently reduced compared to the CTRL, whereas after 24 h, no significant changes are observed.

A completely different trend was observed for protein expression: 24 h after treatment, significant increases in the protein expression levels of both VEGF (Figure 5B) and p-eNOS (Ser1177) (Figure 5D) were detected relative to CTRL. After 48 h, a significant reduction in VEGF protein levels was observed in OsteoBiol^®^ GTO^®^-treated cells compared to CTRL, whereas no significant changes in p-eNOS (Ser1177) levels were observed between treated samples and CTRLs.

### 3.5. OsteoBiol^®^ GTO^®^ Promotes Endothelial Tube Formation

**Figure 6:**
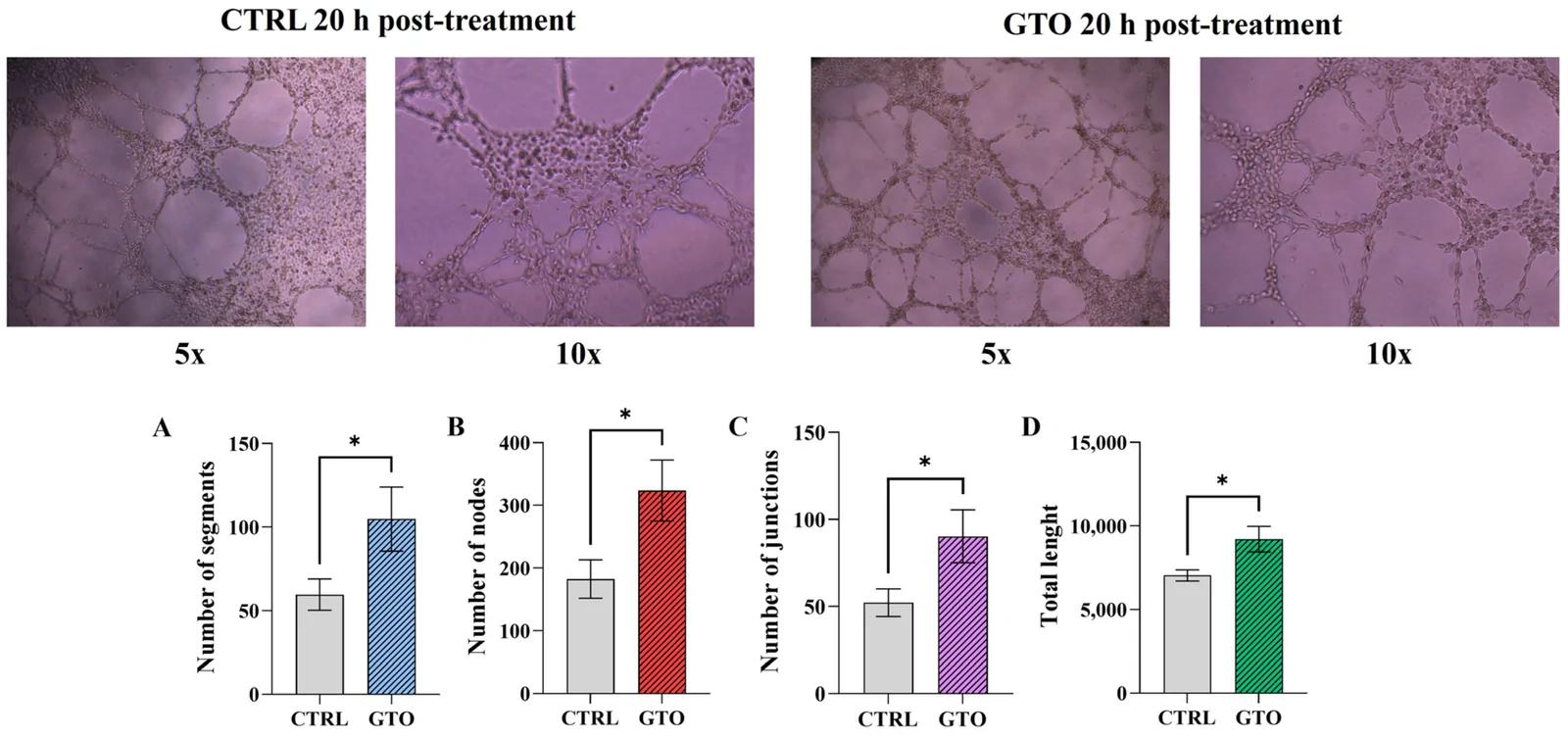
Upper panel: Tube formation assay on EA.hy926 cells cultured on a Matrigel-coated 96-well plate after 20 h of treatment with OsteoBiol^®^ GTO^®^ OS 10 mg/mL. On the left: CTRL cells. On the right: OsteoBiol^®^ GTO^®^-stimulated cells. Magnifications: 5× and 10×. One representative image from three independent experiments is shown for each experimental condition. Lower panel: (**A**) densitometric analysis of the tube formation assay reporting segments, (**B**) nodes, (**C**) junctions, and (**D**) total length (carried out using the Angiogenesis Analyzer plugin in ImageJ). Values represent means ± SD of three independent experiments. Unpaired *t*-test, CTRL vs. OsteoBiol^®^ GTO^®^: * *p* < 0.05.

The tube formation assay on Matrigel-coated plates was performed to evaluate the ability of endothelial cells exposed to OsteoBiol^®^ GTO^®^ to form capillary-like structures. 20 h after seeding, appreciable tubule formation was observed in cells treated with OsteoBiol^®^ GTO^®^ compared to CTRL (Figure 6).

These findings were supported by densitometric analysis, which showed that treatment with OsteoBiol^®^ GTO^®^ produced a statistically significant increase in the number of segments (Figure 6A), nodes (Figure 6B), junctions (Figure 6C), and total length (Figure 6D) compared to CTRL.

## 4. Discussion

An ideal socket filler biomaterial should not only act as a pro-osteogenic material but also appropriately support neo-vascularization, since an efficient blood supply is widely recognized as essential for tissue regeneration [20,25,26,27]. Given published data demonstrating an indirect pro-angiogenic effect of OsteoBiol^®^ GTO^®^ on different cell types, [15,16], the aim of the present work was to better identify the molecular events triggered by OsteoBiol^®^ GTO^®^ that underlie the promotion of angiogenesis when endothelial cells are directly stimulated.

First, the concentration of OsteoBiol^®^ GTO^®^ capable of stimulating endothelial cells and the most appropriate preparation procedure have been established. Previously published studies reported a wide range of OsteoBiol^®^ GTO^®^ concentrations and preparation methods. In particular, the in vitro models proposed in the literature focused on the osteogenic potential of OsteoBiol^®^ GTO^®^ in cells of the dental compartment, such as dental pulp stem cells [17,18], osteoblasts [14], PDL cells [15], and human gingival cells [16], and lacked endothelial cells. Thus, the only available data on OsteoBiol^®^ GTO^®^ and endothelial cells were based on the ability of dental bone compartment cells, after OsteoBiol^®^ GTO^®^ stimulation, to release pro-angiogenic factors, thereby stimulating endothelial cells [15,16]. Therefore, because the present model specifically investigates the direct effects of OsteoBiol^®^ GTO^®^ on endothelial cells, a new screening was needed to identify the most suitable concentrations for eliciting measurable biological responses. Furthermore, regarding the dispensation of OsteoBiol^®^ GTO^®^ in in vitro models, previously published papers reported that OsteoBiol^®^ GTO^®^ was directly applied to cells [14]. Conversely, in the current study, a soaking preparation approach was adopted, as this dispensation mode more closely mimics the clinical effect of OsteoBiol^®^ GTO^®^. Indeed, when used in vivo, OsteoBiol^®^ GTO^®^ is placed within the extraction socket, where it remains in close contact with the alveolar bone. Under these conditions, the biomaterial primarily interacting with the endothelial compartment consists of type I and type III collagen, mainly released from the collagenated OsteoBiol^®^ TSV Gel [28].

Based on the results of this study, after 24 and 48 h of treatment, regardless of the soaking type, 20 mg/mL impaired endothelial cell viability. This effect is presumably due to the high collagen concentration released by OsteoBiol^®^ GTO^®^; indeed, it has been reported elsewhere that high collagen concentration or density, such as in collagenated gel, can reduce cellular oxygen perfusion due to the compactness of collagen fibers [29,30]. Considering the sensitivity of endothelial cells to environmental changes, especially to oxygen supply [31,32], it is plausible to hypothesize that the high concentration of OsteoBiol^®^ GTO^®^ (20 mg/mL), probably through reduced oxygen perfusion, could affect cell viability. Additionally, 1 mg/mL induced a slight reduction in cell viability. However, this effect is unlikely to be due to OsteoBiol^®^ GTO^®^, as the higher concentrations tested (5 and 10 mg/mL) did not induce any variation in cell viability. A more consistent hypothesis for this result is a cellular adaptive response: the lower concentration (1 mg/mL) is insufficient to confer a beneficial effect on cell viability, yet it is perceived as foreign material that endothelial cells must adapt to immediately. Therefore, based on the aforementioned reasons, the MTT results allow us to exclude 1 and 20 mg/mL OsteoBiol^®^ GTO^®^. Based on evidence that metabolic activity did not reveal a suitable soaking preparation method for further experiments, it was decided to proceed by evaluating the collagen released into the medium. Indeed, although a previous study by Jeanneau and colleagues [15] demonstrated that OsteoBiol^®^ GTO^®^ releases a significant amount of collagen compared to other biomaterials, no published papers currently demonstrate which soaking procedure promotes the highest collagen release. The present data demonstrate, for the first time, that collagen release from OsteoBiol^®^ GTO^®^ is dose-dependent: the higher the OsteoBiol^®^ GTO^®^ concentration, the greater the amount of collagen detected. Indeed, 20 mg/mL released the highest amount of collagen in both OS and CS procedures, confirming the previous hypothesis that the medium has a high collagen density. Furthermore, it was demonstrated for the first time that at the same concentration of OsteoBiol^®^ GTO^®^, the OS procedure consistently shows higher collagen levels than CS. This result could be explained by the possibility that the centrifugation procedure causes a slight loss of collagen. Additionally, the CS procedure appears, compared with the OS, to be less similar to the in vivo clinical situation. The DS preparation method did not confer any advantage over OS or CS in cell viability or collagen release; thus, the authors excluded it from subsequent analyses. Collectively, MTT results and collagen detection suggested 10 mg/mL OS as the best combination for further evaluations.

As already mentioned, an efficient blood supply is essential for tissue regeneration, including bone tissue [19]. Based on this, the authors investigated whether direct contact between endothelial cells and OsteoBiol^®^ GTO^®^ OS could trigger a pro-angiogenic effect. Given that collagen is likely the predominant bioactive component released during soaking and to which endothelial cells are exposed, it is plausible to hypothesize that it is the main driver of the pro-angiogenic effect. Indeed, collagen can be recognized by endothelial cell surface receptors, including Discoidin Domain Receptors (DDRs) or integrin α1β2, thereby activating intracellular signaling cascades involving FAK, ERK, NF-κB, and many others, ultimately leading to a pro-angiogenic response that may include COX-2 transcription [33,34,35]. COX-2, in turn, converts arachidonic acid to prostaglandin H2, which is rapidly converted to PGE2. PGE2 is widely recognized as a pro-inflammatory mediator [36]; however, in the vascular compartment, it also functions as a pro-angiogenic factor. Indeed, endothelial cell-derived PGE2 acts in both autocrine and paracrine manners, binding G protein-coupled EP receptors, with EP2 and EP4 predominantly expressed on endothelial cells [37,38]. When appropriately activated, EP2/EP4 stimulate the synthesis of pro-angiogenic factors, such as VEGF, e-NOS, and others [39,40]. One possible signaling pathway activated in this system is the COX-2/PGE2/VEGF pathway: indeed, within 6 h of treatment, COX-2 protein expression increases as an early signal. In parallel, increased PGE2 secretion is observed: it can be assumed that newly synthesized COX-2 within 6 h of treatment is immediately catabolically active in producing PGE2. Consistent with an early pro-angiogenic signal rather than a pro-inflammatory response, PGE2 levels returned to baseline after 24 h of treatment. This effect may be attributable either to a COX-2-mediated negative feedback mechanism or to enhanced COX-2 degradation, as COX-2 protein levels also reverted to those observed in CTRL. VEGF is one of the main early pro-angiogenic factors that stimulate new blood vessel formation [41]. In angiogenesis, activation of EP2/EP4 endothelial cell receptors induces VEGF synthesis [39,40,42]. The temporal profile of the present results suggests a possible involvement of the COX-2/PGE2 axis in VEGF regulation. Indeed, an initial increase in COX-2 and PGE2 levels is observed, followed by increased VEGF protein expression. The reduction in VEGF mRNA levels after 6 h of treatment suggests that the COX-2/PGE2 axis primarily targets the stability of VEGF mRNA and its translational efficiency, rather than transcription. The same profile is observed for e-NOS protein expression: it has been reported that depletion of the EP4 receptor in endothelial cells reduces NO production in blood vessels due to a lack of activation of e-NOS through phosphorylation at ser1177 [43]. In the model used in this study, endothelial cells’ exposure to OsteoBiol^®^ GTO^®^ can induce activation of e-NOS (Ser1177) within 24 h, with no effect on transcription, suggesting a role for OsteoBiol^®^ GTO^®^ downstream of mRNA synthesis without affecting gene expression. The confirmation of an early OsteoBiol^®^ GTO^®^-mediated pro-angiogenic effect, together with stimulation of the COX-2/PGE2 axis at early time points and increased VEGF and p-eNOS protein expression, is supported by the observation that after 48 h of treatment, both protein expressions decrease, underscoring their role as early pro-angiogenic factors. Additional evidence of OsteoBiol^®^ GTO^®^’s pro-angiogenic role is provided by the tube formation result: after 20 h of treatment, cells exposed to the OS exhibited a well-organized network of vessel-like structures. This may indicate that although VEGF becomes detectable after 24 h, it can likely exert functional effects on vascular structure formation earlier, as evidenced by a well-organized tube network. However, this process may be partially VEGF-dependent, and additional factors, such as activated eNOS, may also be involved. For this reason, further focused studies are still required to confirm the hypothesis.

## 5. Conclusions

OsteoBiol^®^ GTO^®^ is an innovative xenograft biomaterial for socket preservation after tooth extraction, with well-documented pro-osteogenic effects in in vitro and in vivo systems. In addition to the information already available, the present study demonstrated a promising pro-angiogenic effect of OsteoBiol^®^ GTO^®^ when it is in close contact with endothelial cells. Despite additional studies being necessary to validate the proposed mechanism of action, these data demonstrated for the first time that: (i) OsteoBiol^®^ GTO^®^ can release collagen, and the soaking preparation can influence the final amount of detectable collagen. Therefore, the most appropriate procedure for preparing OsteoBiol^®^ GTO^®^ for in vitro models is the OS; (ii) the study provided the first direct evidence that OsteoBiol^®^ GTO^®^ exerts a significant pro-angiogenic effect on an in vitro model of endothelial cells, with significant modulation of the COX-2/PGE2/VEGF signaling axis, presumably triggered by collagen released in the OS; (iii) consistent with the suggested pro-angiogenic effect, tube-like structure formation confirmed a promising use of OsteoBiol^®^ GTO^®^ as a pro-angiogenic material. These findings add a new dimension to the understanding of OsteoBiol^®^ GTO^®^’s biological activity beyond its established osteoconductive properties, with important implications for its clinical use in socket preservation and guided bone regeneration.

## Figures and Tables

**Table 1:** Primary antibodies used for Western blot and corresponding dilutions.

Target Protein	Primary Antibody (Host Specie/Type)	Supplier	Dilution
COX-2	Mouse monoclonal	Santa Cruz Biotechnology (Dallas, TX, USA)	1:200
VEGF	Mouse monoclonal	ThermoFischer Scientific (Waltham, MA, USA)	1:200
e-NOS	Rabbit polyclonal	Santa Cruz Biotechnology (Dallas, TX, USA)	1:200
p-eNOS (Ser1177)	Rabbit monoclonal	Cell Signaling Technology (Danvers, MA, USA)	1:1000
β-Actin	Mouse monoclonal	Invitrogen (Waltham, MA, USA)	1:5000
Vinculin	Rabbit polyclonal	Invitrogen (Waltham, MA, USA)	1:1000

**Table 2:** Primer sequences for quantitative Polymerase Chain Reaction (qPCR).

Target Gene	Sequence (5′-3′)
VEGF-FW	CCGGTATAAGTCCTGGAGCG
VEGF-RW	GCAACGCGAGTCTGTGTTTT
eNOS-FW	GTGTCCCTCGAACACGAGAC
eNOS-RW	AGTGGGTCTGAGCAGGAGAT
GAPDH-FW	GGGTGTGAACCATGAGAAGTA
GAPDH-RW	ACTGTGGTCATGAGTCCTTC

## Data Availability

The original contributions presented in this study are included in the article. Further inquiries can be directed to the corresponding author.

## Abbreviations

The following abbreviations are used in this manuscript:

GBR	Guided Bone Regeneration
PDL	Periodontal Ligament
hDPSCs	Human Dental Pulp Stem Cells
HUVECs	Human Umbilical Vein Endothelial Cells
COX-2	Cyclooxygenase-2
PGE2	Prostaglandin E-2
VEGF	Vascular Endothelial Growth Factor
DMEM	Dulbecco’s Modified Eagle Medium
FBS	Fetal Bovine Serum
OS	Original Soaking
CS	Centrifuged Soaking
DS	Diluted Soaking
MTT	3-(4,5-dimethylthiazol-2-yl)-2,5-diphenyltetrazolium bromide
DMSO	Dimethyl Sulfoxide
CTRL	Untreated Cells
OD	Optical Density
HRP	Horseradish Peroxidase
RT	Reverse Transcription
PCR	Polymerase Chain Reaction
qPCR	Quantitative Polymerase Chain Reaction
SD	Standard Deviation
ANOVA	Analysis of Variance
eNOS	Endothelial Nitric Oxide Synthase
DDRs	Discoidin Domain Receptors
